# Characteristics and short-term outcomes of patients with esophageal cancer with unplanned intensive care unit admissions: a retrospective cohort study

**DOI:** 10.5935/0103-507X.20200041

**Published:** 2020

**Authors:** Isabel Cristina Lima de Freitas, Dryelen Moreira de Assis, Cristina Prata Amendola, Diana da Silva Russo, Ana Paula Pierre de Moraes, Pedro Caruso, Antonio Paulo Nassar Júnior

**Affiliations:** 1 A. C. Camargo Cancer Center - São Paulo (SP), Brasil.; 2 Hospital de Câncer de Barretos - Barretos (SP), Brasil.; 3 Hospital Moinhos de Vento - Porto Alegre (RS), Brasil.; 4 Hospital de Câncer do Maranhão “Tarquínio Lopes Filho” - São Luís (MA), Brasil.

**Keywords:** Critical care, Critical care outcomes, Esophageal neoplasms, Respiration, artificial, Mortality, Prognosis, Epidemiology, Cuidados críticos, Resultados de cuidados críticos, Neoplasias esofágicas, Respiração artificial, Mortalidade, Prognóstico, Epidemiologia

## Abstract

**Objective:**

To depict the clinical presentation and outcomes of a cohort of critically ill patients with esophageal cancer.

**Methods:**

We carried out a multicenter retrospective study that included patients with esophageal cancer admitted to intensive care units with acute illness between September 2009 and December 2017. We collected the demographic and clinical characteristics of all included patients, as well as organ-support measures and hospital outcomes. We performed logistic regression analysis to identify independent factors associated with in-hospital mortality.

**Results:**

Of 226 patients included in the study, 131 (58.0%) patients died before hospital discharge. Squamous cell carcinoma was more frequent than adenocarcinoma, and 124 (54.9%) patients had metastatic cancer. The main reasons for admission were sepsis/septic shock and acute respiratory failure. Mechanical ventilation (OR = 6.18; 95%CI 2.86 - 13.35) and metastatic disease (OR = 7.10; 95%CI 3.35 - 15.05) were independently associated with in-hospital mortality.

**Conclusion:**

In this cohort of patients with esophageal cancer admitted to intensive care units with acute illness, the in-hospital mortality rate was very high. The requirement for invasive mechanical ventilation and metastatic disease were independent prognostic factors and should be considered in discussions about the short-term outcomes of these patients.

## Abstract (pt)

**Objetivo:**

Mostrar o quadro clínico e os desfechos de uma coorte de pacientes críticos com câncer esofágico.

**Métodos:**

Conduzimos um estudo multicêntrico retrospectivo que incluiu pacientes com câncer esofágico admitidos a unidades de terapia intensiva em razão de doença aguda entre setembro de 2009 e dezembro de 2017. Colhemos os dados demográficos e as características clínicas de todos os pacientes incluídos, assim como as medidas de suporte a órgãos e os desfechos no hospital. Realizamos uma análise de regressão logística para identificar os fatores associados de forma independente com mortalidade hospitalar.

**Resultados:**

Dentre os 226 pacientes incluídos no estudo, 131 (58,0%) faleceram antes de receber alta hospitalar. O carcinoma espinocelular foi mais frequente do que o adenocarcinoma, e 124 (54,9%) pacientes tinham câncer metastático. As principais razões para admissão foram sepse/choque séptico e insuficiência respiratória aguda. Uso de ventilação mecânica (RC = 6,18; IC95% 2,86 - 13,35) e doença metastática (RC = 7,10; IC95% 3,35 - 15,05) tiveram associação independente com mortalidade hospitalar.

**Conclusão:**

Nesta coorte de pacientes com câncer esofágico admitidos à unidades de terapia intensiva em razão de doença aguda, a taxa de mortalidade hospitalar foi muito elevada. A necessidade de utilizar ventilação mecânica invasiva e a presença de doença metastática foram fatores independentes de prognóstico e devem ser levados em conta nas discussões a respeito dos desfechos destes pacientes em curto prazo.

## INTRODUCTION

Esophageal cancer is among the most common cancers worldwide. Its 5-year survival rate, although still poor, has improved considerably in recent years.^(1,2)^ Treatment typically involves chemotherapy, radiotherapy, and extensive surgery, all of which are associated with severe complications. Postoperative and clinical complications are associated with increased mortality in patients with esophageal cancer.^(3-5)^

Intensive care unit (ICU) admission is common among patients with cancer. Although many studies have examined epidemiological patterns and outcomes among critically ill patients with cancer,^(6-8)^ few studies have addressed whether specific types of cancer have different presentations and outcomes. For example, although cancer status and complications have not been associated with short-term mortality in the majority of studies of critically ill patients,^(7)^ disease stage has been identified as a prognostic factor in patients with advanced lung cancer^(9)^ and head and neck cancer.^(10)^

More than one-quarter of all patients with esophageal cancer are admitted to the ICU during the first 2 years after diagnosis.^(11)^ However, most studies of these patients have examined only postoperative outcomes following esophagectomy.^(3,4)^ Therefore, little is known about the characteristics and outcomes of patients with esophageal cancer who have unplanned ICU admissions due to acute illness.

The aims of this study were to depict the clinical presentation and outcomes of a cohort of critically ill patients with esophageal cancer and to identify the risk factors associated with in-hospital mortality in these patients.

## METHODS

In this retrospective cohort study, the medical records of patients with esophageal cancer who were admitted to four ICUs in Brazil between September 2009 and December 2017 were examined. Three hospitals were dedicated cancer centers (A.C. Camargo Cancer Center, *Hospital de Câncer de Barretos* and *Hospital de Câncer do Maranhão “Dr. Tarquinio Lopes Filho”*), and one was a general hospital with a high volume of cancer patients (*Hospital Moinhos de Vento*). The study was approved by the ethics committees of all participating centers. Due to the observational and retrospective nature of the study, the requirement for informed patient consent was waived. We followed the STROBE (STrengthening the Reporting of OBservational studies in Epidemiology) guidelines for the reporting of observational studies.^(12)^

The inclusion criteria for this study were a confirmed diagnosis of esophageal cancer, age ≥ 18 years, and admission for medical or urgent surgical reasons. Only the first ICU admission was taken into account. We excluded patients admitted for elective surgery and those transferred to other hospitals before discharge.

We collected clinical data obtained at admission and data on clinical outcomes at the time of hospital discharge from patients’ electronic medical records. We collected the following data obtained at admission: age, sex, Simplified Acute Physiology Score (SAPS) 3, Sequential Organ Failure Assessment (SOFA) score, Charlson Comorbidity Index (CCI), Eastern Cooperative Oncology Group (ECOG) performance status, histological type (adenocarcinoma or squamous cell carcinoma), cancer stage, and source of and reason for admission. We also collected the following data regarding ICU and hospital stays and complications: cancer-related complications (tumor mass, bleeding, stenosis, and fistulae), delirium, organ support during ICU stay (vasopressors, mechanical ventilation, and renal replacement therapy), and lengths of ICU and hospital stays. Since points originating from tumor characteristics impact the CCI calculation, we reported a modified version of the CCI that did not take into account points from cancer characteristics. The primary outcome was in-hospital mortality.

### Statistical analysis

Continuous variables are presented as medians and interquartile ranges, and categorical variables are presented as absolute numbers and percentages. Univariate analysis was performed to compare data from patients who survived and those who died during hospitalization using the Mann-Whitney or chi-squared test, as appropriate. We did not adjust for multiple comparisons in the univariate analyses.

We performed logistic regression analysis to identify independent prognostic variables among seven clinical variables defined a priori (modified CCI, performance status [categorized as ECOG 0 - 1 *versus* ECOG 2 - 4], metastatic disease [cancer stage IV], occurrence of *delirium*, need for mechanical ventilation, vasopressor use, and renal replacement therapy during ICU stay). First, we evaluated collinearity by measuring the variation inflation factor (VIF). We considered a VIF > 2 as diagnostic of multicollinearity. In the case of multicollinearity, we included only the more clinically relevant variable. There were no missing values for outcomes, but there were missing values for cancer status (and, consequently, CCI) in four patients and for performance status in two. We did not impute missing values and proceeded to a complete-case analysis. The odds ratio (OR) and 95% confidence interval (95%CI) were calculated for each variable included in the model. Model calibration was assessed by the Hosmer-Lemeshow (H-L) goodness-of-fit test. A p value > 0.05 for this test was indication of good calibration. All data were analyzed with Statistical Package for Social Science (SPSS, IBM Corporation, Armonk, NY, USA) version 21.

## RESULTS

**Figure 1:**
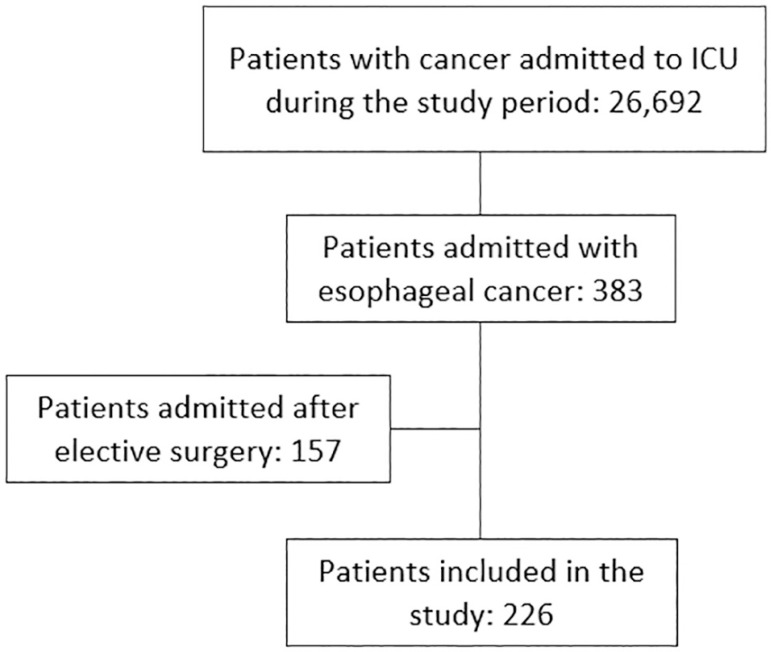
Study flowchart. ICU - intensive care unit.

This study included 226 patients with esophageal cancer admitted to the ICU between September 2009 and December 2017 (Figure 1). Table 1 shows the patients’ characteristics. Squamous cell carcinoma was more frequent than adenocarcinoma, and most patients had advanced cancer. The patients were admitted predominantly from emergency rooms and wards, and the main reasons for admission were sepsis/septic shock and acute respiratory failure. Cancer-related complications were common.

**Table 1:** Characteristics of patients with esophageal cancer admitted to intensive care units due to acute illness according to vital status at hospital discharge

Variable	Alive (n = 95)	Dead (n = 131)	p value
Age (years)	61 (55 - 68)	64 (56 - 72)	0.01
Male	81 (85.3)	97 (74.0)	0.04
Histological type*			0.83
Squamous cell carcinoma	70 (73.7)	101 (77.1)	
Adenocarcinoma	24 (25.3)	29 (22.1)	
Cancer stage			< 0.01
II or III	60 (63.1)	42 (32.1)	
IV	35 (36.8)	89 (67.9)	
Modified CCI	2 (0 - 4)	2 (0 - 3)	0.52
ECOG status			0.07
0 - 1	47 (49.5)	49 (37.4)	
SAPS 3	60.5 (55 - 71.25)	69.5 (61.75 - 79.25)	< 0.01
SOFA score	2 (1 - 5)	4 (2 - 7)	< 0.01
Source of admission			0.03
Emergency room	38 (40.0)	48 (36.6)	
Ward	42 (44.2)	76 (58.0)	
Surgical room	11 (11.6)	6 (4.6)	
Another hospital	4 (4.2)	1 (0.8)	
Reason for admission			0.12
Sepsis/septic shock	28 (39.4)	44 (33.6)	
Acute respiratory failure	20 (21.1)	46 (35.1)	
Neurological disorders	7 (7.4)	7 (7.4)	
Cardiovascular disorders	12 (12.6)	6 (4.6)	
Hemorrhage	5 (5.3)	3 (2.3)	
Cancer-related complications			
Stenosis	9 (9.5)	12 (9.2)	0.94
Fistulae	17 (17.9)	28 (21.4)	0.52
Tumor mass	11 (11.6)	34 (26.0)	< 0.01
Bleeding	10 (10.5)	12 (9.1)	0.73
Clinical complications			
Pneumonia	33 (34.7)	50 (38.2)	0.64
Atrial fibrillation	13 (13.7)	31 (23.7)	0.06
*Delirium*	12 (12.6)	30 (22.9)	0.05
Organ support			
Mechanical ventilation	22 (23.2)	75 (57.3)	< 0.01
Vasopressors	32 (33.7)	63 (48.1)	0.02
Renal replacement therapy	4 (4.2)	10 (7.6)	0.29
Length of ICU stay (days)	3 (2 - 6)	3 (1 - 6)	0.72
Length of hospital stay (days)	13 (8 - 26)	3 (1 - 12)	< 0.01

CCI - Charlson Comorbidity Index; ECOG - Eastern Cooperative Oncology Group; SAPS - Simplified Acute Physiology Score; SOFA - Sequential Organ Failure Assessment; ICU - intensive care unit.

* Two missing values. Results expressed as median (interquartile range) or n (%).

In total, 131 (58.0%) patients died before hospital discharge. Patients who died had higher SAPS 3 and SOFA scores at admission, higher comorbidity burdens, more metastatic disease, and increased needs for mechanical ventilation and vasopressors.

There was no multicollinearity among the chosen variables (Table 2). Mechanical ventilation (OR = 6.18; 95%CI, 2.86 - 13.35) and metastatic disease (OR = 7.10; 95%CI, 3.35 - 15.05) were independently associated with in-hospital mortality (Table 3). The model was well calibrated (H-L = 7.33, p = 0.50).

**Table 2:** Variation inflation index of the selected variables to be included in the logistic regression model

Variable	VIF
ECOG	1.07
Metastastic disease	1.15
Modified CCI	1.26
*Delirium*	1.05
Mechanical ventilation	1.26
Vasopressors	1.26
Renal replacement therapy	1.14

VIF - variation inflation index; ECOG - Eastern Cooperative Oncology Group; CCI - Charlson comorbidity index.

**Table 3:** Logistic regression results for risk factors independently associated with in-hospital mortality

Variable	OR	95%CI
ECOG 2 - 4	1.34	0.94 - 1.92
Metastatic disease	7.10	3.35 - 15.05
Modified CCI	0.88	0.74 - 1.05
*Delirium*	1.65	0.71 - 3.82
Vasopressor use	1.34	0.65 - 2.76
Mechanical ventilation	6.18	2.86 - 13.35
Renal replacement therapy	1.25	0.32 - 4.86

OR - odds ratio; 95%CI - 95% confidence interval; ECOG - Eastern Cooperative Oncology Group; CCI - Charlson comorbidity index.

## DISCUSSION

This study showed that patients with esophageal cancer admitted to the ICU for acute illness were severely ill and had a high in-hospital mortality rate. Mechanical ventilation and metastatic disease were independently associated with in-hospital mortality.

Although many studies have addressed the characteristics and outcomes of critically ill patients with solid cancers^(7)^ as well as patients with esophageal cancer admitted to the ICU after elective esophagectomy,^(3,4)^ to our knowledge, no study has focused on patients with esophageal cancer admitted with acute illness. In our cohort, such patients had a higher mortality rate than critically ill patients with solid cancers in general.^(7)^ Our results are comparable to those reported for patients with advanced lung cancer^(9)^ and head and neck cancer^(10)^ admitted to ICUs due to acute illness. In a Dutch study of short- and long-term outcomes of patients admitted to the ICU with different types of cancer, esophageal cancer was associated with a 30-day survival rate of 93%.^(11)^ However, the authors did not differentiate between patients admitted for elective and nonelective reasons. More than one-quarter of patients with esophageal cancer in this cohort were admitted to the ICU during follow-up, and many may have been selected patients with favorable therapeutic prospects.^(11)^ In another study of the same cohort, upper gastrointestinal cancer was independently associated with in-hospital mortality among patients with unplanned admissions.^(13)^ These results are in accordance with those of other studies suggesting that ICU admission for acute illness is associated with 50% - 70% greater mortality than is admission for elective surgery among patients with cancer.^(8,14)^

Mechanical ventilation is a well-known risk factor for mortality in patients with cancer. The majority of the studies included in a systematic review examining the prognosis of ICU-admitted patients with solid cancer showed that the need for mechanical ventilation was associated with higher mortality rates.^(7)^ In a Brazilian study, 25% of all patients with cancer admitted to ICUs required invasive mechanical ventilation, and their in-hospital mortality rate was 73%.^(15)^ In our study, 44% of patients required mechanical ventilation during their ICU stay, and their in-hospital mortality rate was 76%. Therefore, as in patients with other types of cancer, the requirement for mechanical ventilation is a marker of dismal prognosis in patients with esophageal cancer.

On the other hand, cancer characteristics *per se* are not consistently associated with worse short-term prognoses. In the systematic review conducted by Puxty et al.,^(7)^ findings from a minority of studies suggested that advanced or metastatic cancer was associated with higher ICU, in-hospital, and 30-day mortality. However, metastatic disease has been independently associated with short-term mortality in patients with advanced lung cancer^(9)^ and those with head and neck cancer.^(10)^ It also seems to be a marker of severity in ICU-admitted patients with esophageal cancer.

Our study has some important limitations. First, as it was a retrospective cohort study, so no causal inference could be drawn. Additionally, it was prone to bias due to data collection. Second, although it involved multiple centers, the study included only Brazilian hospitals with high volumes of patients with cancer. Thus, our results may not be widely generalizable. Third, we did not have access to data on decisions to limit support, which could have influenced the mortality rate of these severely ill patients. Importantly, since deceased patients had a median ICU and hospital stay of only three days, it is possible that withholding life-sustaining therapies was decided early during the ICU stay, taking into consideration some specific patient characteristics, such as metastatic disease, and, therefore, this may have created a self-fulfilling prophecy bias.

## CONCLUSION

In this cohort of patients with esophageal cancer admitted to intensive care units with acute illness, the in-hospital mortality rate was very high. The requirement for invasive mechanical ventilation and metastatic disease were independent prognostic factors and should be taken into account in discussions about the short-term outcomes of patients with esophageal cancer who are admitted to intensive care units due to acute illness.
